# Prevalence of colistin resistance of *Klebsiella pneumoniae* isolates in Iran: a systematic review and meta-analysis

**DOI:** 10.1186/s12941-022-00520-8

**Published:** 2022-06-28

**Authors:** Negar Narimisa, Forough Goodarzi, Shirin Bavari

**Affiliations:** 1 Department of Microbiology, School of Medicine, Iran University of Medical Sciences, Tehran, Iran; 2 Department of Bacteriology, Faculty of Medical Sciences, Tarbiat Modares University, Tehran, Iran

**Keywords:** *Klebsiella pneumoniae*, Antibiotic resistance, Colistin, Meta analysis, CMA

## Abstract

**Objective:**

*Klebsiella pneumoniae* is a gram-negative pathogen common cause of nosocomial infections. Colistin is a last resort antibiotic to treat infections caused by *K. pneumoniae*. In recent years, the resistance rate to colistin has increased in *K. pneumoniae*. This study evaluated the prevalence of colistin resistance of *K. pneumoniae* isolates in Iran using a systematic review and meta-analysis.

**Method:**

A systematic search was performed for relevant articles until August 2021 in the following database: PubMed, Scopus, SID and Google Scholar. The pooled prevalence of colistin resistance in clinical *K. pneumoniae* isolates analyzed using Comprehensive Meta-Analysis Software (CMA).

**Results:**

Finally, 19 articles with appropriate criteria were included in the meta-analysis. Our results showed 6.9% of the pooled prevalence of colistin resistance in clinical *K. pneumoniae* isolates in Iran. The results of subgroup analysis demonstrated increase resistance of colistin from 4.8%; (95% CI 1.5–13.9%) in 2013–2018 to 8.2%; (95% CI 3.4–18.6%), in 2019–2021. Also, the results of our study showed a strong association between the carbapenem producing *K. pneumoniae* and increased resistance to colistin.

**Conclusions:**

This study showed a high prevalence of colistin resistance in *K. pneumoniae* isolates. It is recommended that regular evaluation be performed to control colistin resistance.

**Supplementary Information:**

The online version contains supplementary material available at 10.1186/s12941-022-00520-8.

## Introduction

Gram-negative bacterial (GNB) resistance to antimicrobials is increasing worldwide [1]. It is a significant public health problem and causes critical morbidity and mortality in hospitalized patients [2]. There is a relationship between antibiotic resistance and mortality rate of patients and length of hospital stay, and increased treatment costs [3]. Many gram-negative bacteria cause nosocomial infections, among which *Klebsiella pneumoniae* of the Enterobacteriaceae family plays a vital role due to its potent antibiotic resistance [4]. *K. pneumoniae* is a gram-negative, encapsulated, nonmotile, rod-shaped bacterium that is an actual cause of nosocomial infections that can lead to various infections, including respiratory, urinary tract and wound infections [5, 6]. Overuse of antibiotics have led to problems in the treatment of *K. pneumoniae* and limited options available for effective treatment of this bacterial infection [7]. The emergence of MDR *K. pneumoniae* has become very challenging due to their resistance to most antimicrobial drugs [8]. Polymyxin antibiotics such as colistin are one of the few antimicrobial agents that have activity against MDR-GNB and are considered the last line of treatment for MDR-GNB infections [9]. Polymyxins are cationic antimicrobial peptides that target the phosphate portion of the bacterial lipopolysaccharide (LPS), disrupts the negative charge of the outer membrane and causes cell death [10, 11]. Colistin resistance is mainly due to the covalent modification of LPS, resulting in decreased affinity between LPS and colistin [12]. Other possible mechanisms are overexpression of the efflux pump, increased capsule synthesis, and production of colistinase. The two-component system (TCS), consisting of PhoPQ and PmrAB, are regulatory systems that reduce the negative charge of lipid A and the binding affinity of colistin to LPS [13, 14]. Considering the importance of colistin as one of the last lines of treatment and the lack of a meta-analysis article on the resistance of *K. pneumoniae* to colistin, the present study aimed to investigate the prevalence of colistin resistance in *K. pneumoniae* isolates in Iran.

## Material and method

### Research strategy

Systematic research was performed on PubMed, Scopus, SID, and Google Scholar for published studies of prevalence colistin resistance *K. pneumoniae* isolates in Iran. All these databases were searched followed by this search strategy: (“Klebsiella pneumoniae” OR K.pneumoniae ) AND (Resistan* OR suscep*) AND (Colisticin OR “Polymyxin E” OR Colimycin OR  colistin OR colistimethate) AND Iran (Additional file 1).

### Inclusion and exclusion criteria for studies

The following studies were included in our study based on the following criteria: (1) original articles that reported the total number of *K. pneumoniae* isolates and the number of colistin resistant (2) Studies conducted in Iran (3) Studies were written in Persian or English.

Studies with insufficient information, reviews, comments, case report, studies not reporting *K. pneumoniae* isolates separately (total isolates and number of resistance) and studies that report the prevalence of colistin resistance of *K. pneumoniae* in animals were excluded.

### Data extraction

**Fig. 1:**
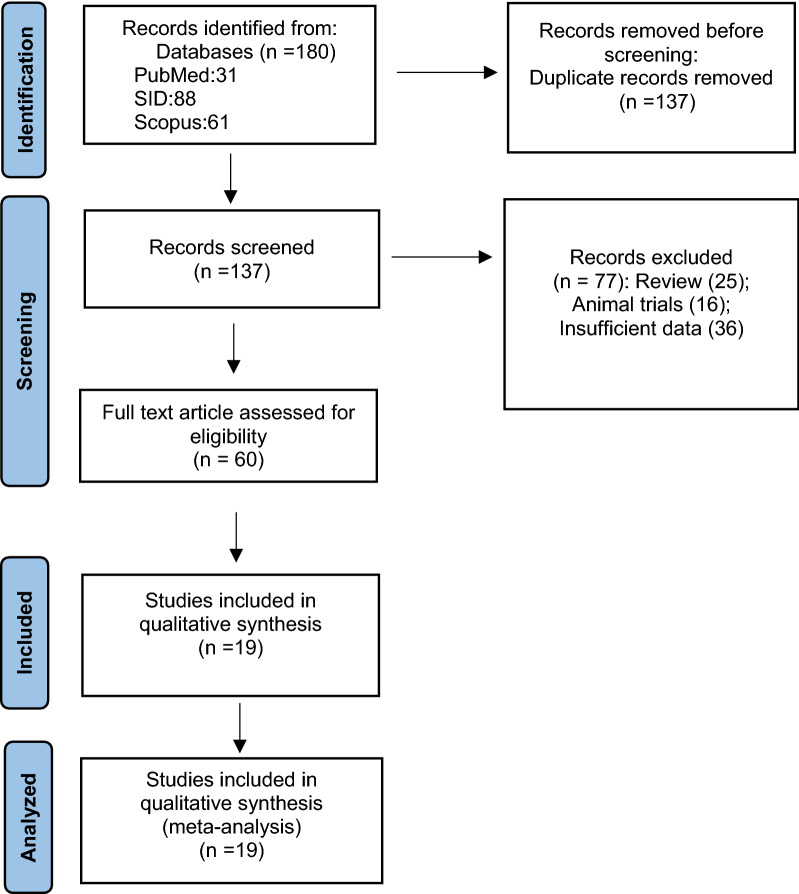
The study Prisma flow diagram

For data extraction, the abstracts and the full texts were independently searched according to the eligibility criteria by two researchers (Goodarzi F, Bavari Sh). The results were reviewed by a corresponding author (Narimia N), and any discrepancies between the researchers were resolved by a consensus and discussion. Data extraction format for the included article was: first author, publication year, study city, number of total isolates, number of colistin resistance isolates, detection method and guidelines for interrupting results (Table 1). This study selection process was presented in a Preferred Reporting Item for Systematic Reviews and Meta-Analyses (PRISMA) flowchart (Fig. 1).

**Table 1:** Characteristics of included studies

First author	Year	City	Method for assay	Guideline	Quality	Number of isolates	Number of resistance
Alizadeh [31]	2021	Isfahan	Broth microdilution	EUCAST	8	240	10
Heidary [32]	2016	Tehran	Disk diffusion	CLSI	7	117	5
Karimi [5]	2021	Hamadan	Broth microdilution	EUCAST	9	83	0
Mamishi [33]	2019	Tehran	–	CLSI	6	6	0
Mirshekar [34]	2020	Tehran	Broth microdilution	CLSI	9	94	20
Moosavian [35]	2019	Ahvaz	Broth microdilution	EUCAST	9	119	26
Saadatian [36]	2018	Tehran	Broth microdilution	CLSI	7	81	14
Shahraki-Zahedani [37]	2016	Zahedan	Disk diffusion	CLSI	6	170	2
Sharahi [38]	2021	Tehran	Broth microdilution	CLSI	7	52	16
Sheikh [39]	2021	Ahvaz	Disk diffusion	CLSI	8	120	2
Vaziri [40]	2017	Kermanshah	Disk diffusion	CLSI	7	57	2
Nejad [41]	2017	Arak	Disk diffusion	CLSI	7	100	0
Sheshblouki [42]	2016	Shiraz	Disk diffusion	CLSI	6	111	4
Darabi [43]	2015	Urmia	Disk diffusion	CLSI	7	182	0
Carbapenemase-producing *K. pneumoniae*							
Bahrami [44]	2021	Isfahan	Disk diffusion	CLSI	7	62	43
Latifi [45]	2020	Bushehr	Broth microdilution	CLSI	7	12	0
Solgi [46]		Isfahan	Broth microdilution	EUCAST	8	74	0
Jafari [47]	2017	Tehran	Disk diffusion	CLSI	7	100	50
Moghimi [48]	2021	Tabriz	Broth microdilution	CLSI	5	5	2

### Study quality assessment

The quality of studies was assessed using standard critical appraisal tools prepared by Joanna Briggs Institute (JBI) [15, 16]. This appraisal checklist for prevalence studies has nine essential questions. These questions focus on appropriate sampling frame, study subject and sufficient data analysis. Each item is graded yes, no or unclear. A score of 1 was given for the answer “yes”, while a score of 0 was given for the answer “no” and “unclear”. Finally, the mean score was independently calculated for each article independently with two authors (Goodarzi F, Bavari Sh) in consultation with a correspond author (Narimisa N). Studies with scores of 5 and above were rated as high quality.

### Data analysis

Data Analysis of the prevalence of colistin resistance clinical *K. pneumoniae* isolates was calculated in Comprehensive Meta-Analysis Software (CMA). Subgroup analyses were done according to the publication year, study city, detection method and type of *K. pneumoniae* isolates. A random-effects model was applied to estimate the pooled prevalence of colistin resistance among clinical *K. pneumoniae* isolates in Iran at 95% CI.

Heterogeneity was checked using I^2^ test statistics. I^2^ ≤ 25% indicated low homogeneity, 25% < I^2^ ≤ 75% indicated moderate heterogeneity, and I^2^ > 75% indicated high heterogeneity. Funnel plot diagrams and Begg’s test were used to assess the existence of publication bias. The results were considered to have a publication bias at *P* < 0.05.

## Results

### Search results

A total of 180 studies were found, and after reviewing and removing duplicates through the Endnote software, 161 studies were excluded as they did not have the inclusion criteria. Finally, only 19 studies were potentially eligible, included in this meta-analysis (Fig. 1). In this study, 1532 isolates from 12 cities of Iran from 2015 to 2021 were examined. Disc diffusion and broth microdilution methods were used to detect antibiotic susceptibility testing of isolates. In 4 articles, the results were interpreted by EUCAST Guideline and in 15 articles by CLSI (Table 1).

**Fig. 2:**
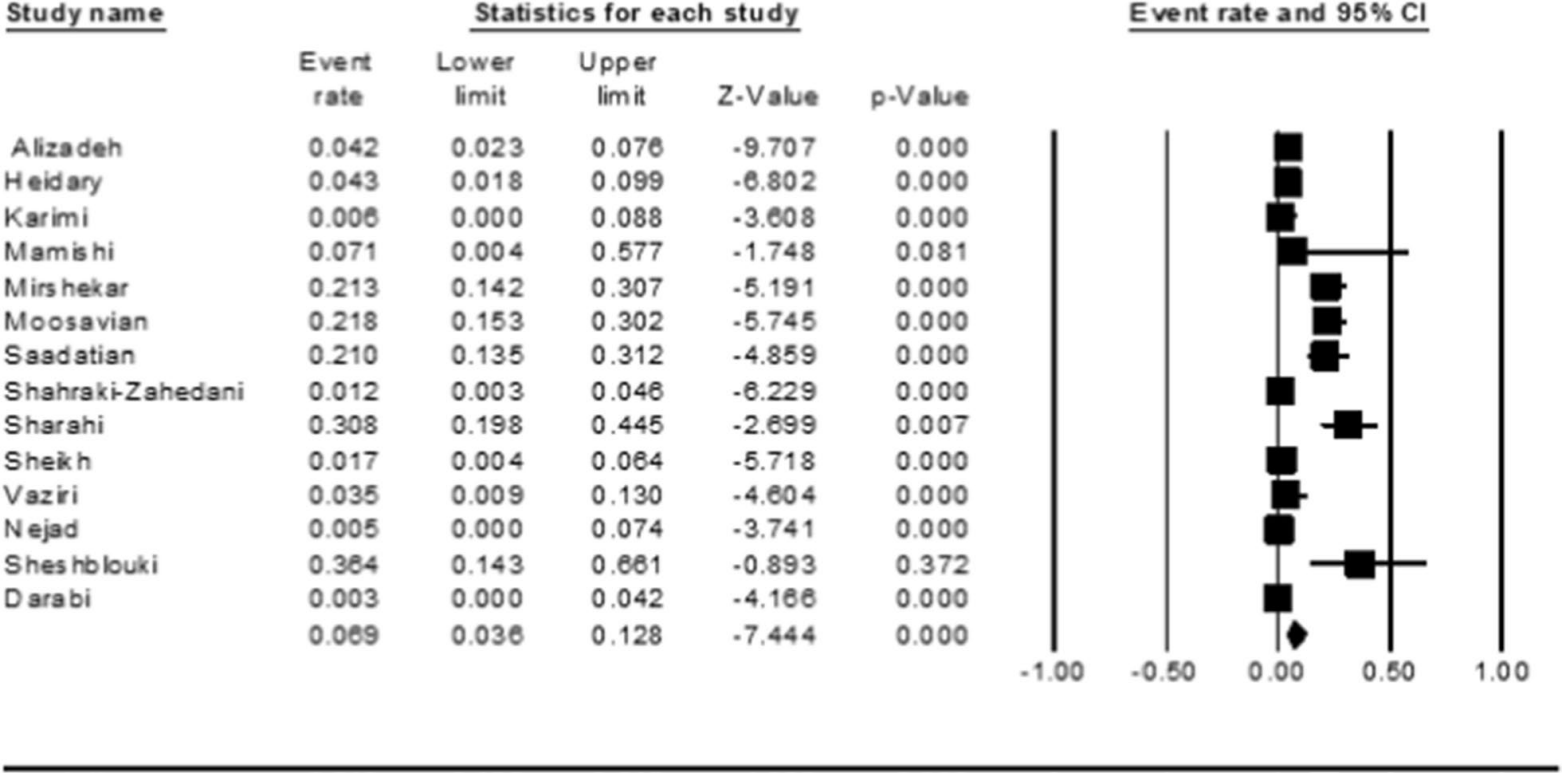
Forest plot of prevalence of colistin resistance of *k. pneumoniae* isolates in Iran

**Fig. 3:**
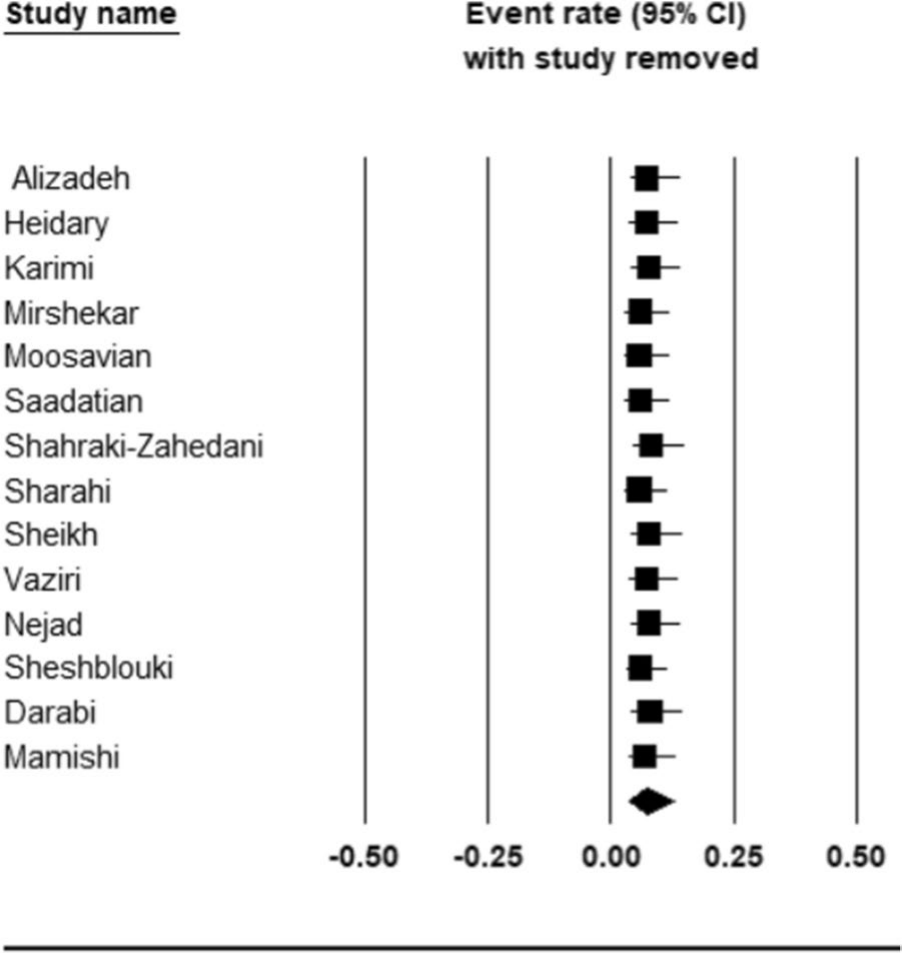
Forest plot of sensitivity analyses

**Fig. 4:**
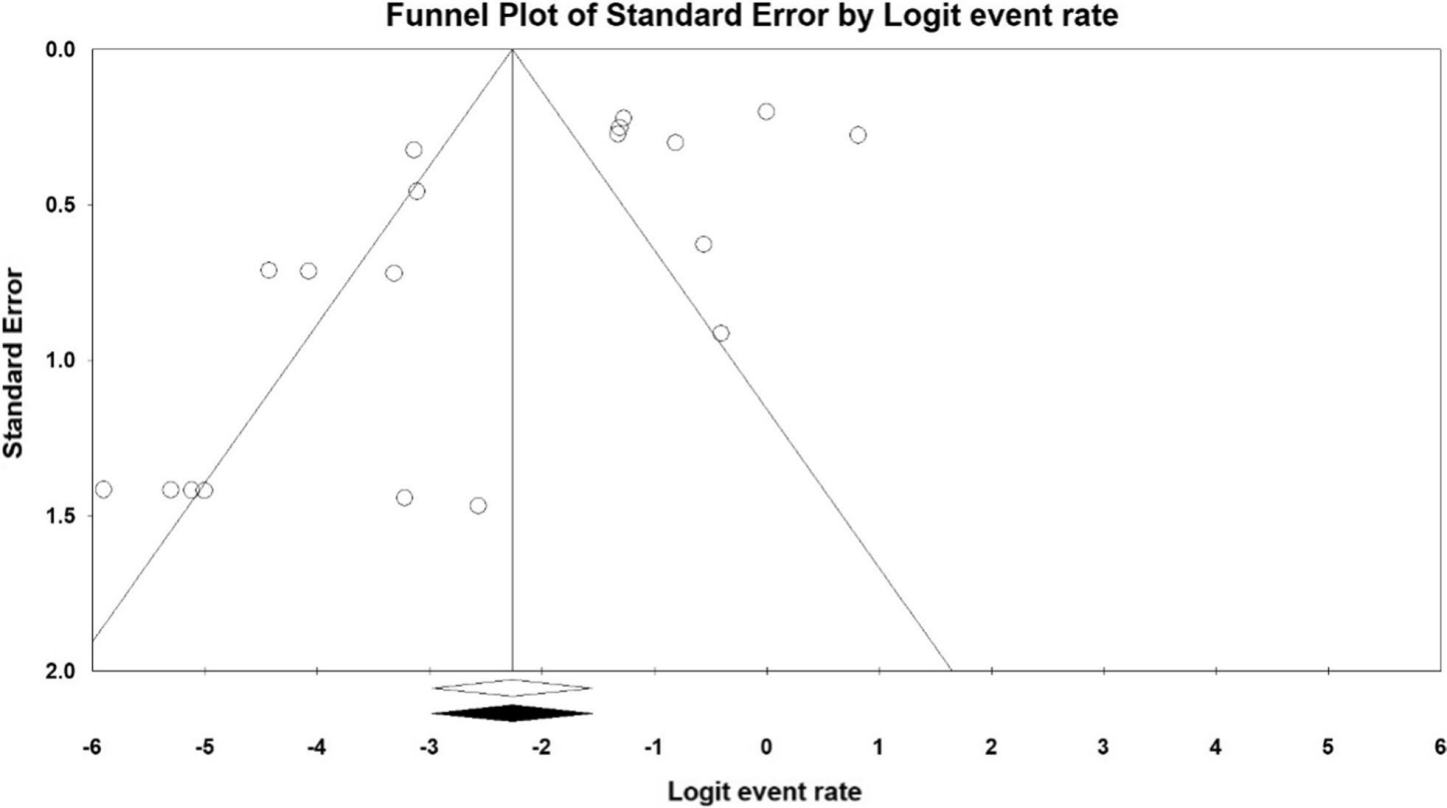
Funnel plot for meta-analysis

The pooled prevalence of colistin resistance in clinical *K. pneumoniae* isolates was estimated at 6.9% (95% CI; 3.6_ 12.8%; I^2^ = 87.12%; *P* < 0.001) (Fig. 2). Sensitivity analyses were performed, and no studies affected the prevalence of colistin-resistant isolates, as shown in the susceptibility analysis’s forest plot (Fig. 3). The result of publication bias was shown in the funnel plot; also, Begg’s tests was used to indicate the extent of bias (*P* = 0.352) (Fig. 4).

### Subgroup meta-analysis

Subgroup analysis was done according to the publication year, city of included studies, and detection method.

**Fig. 5:**
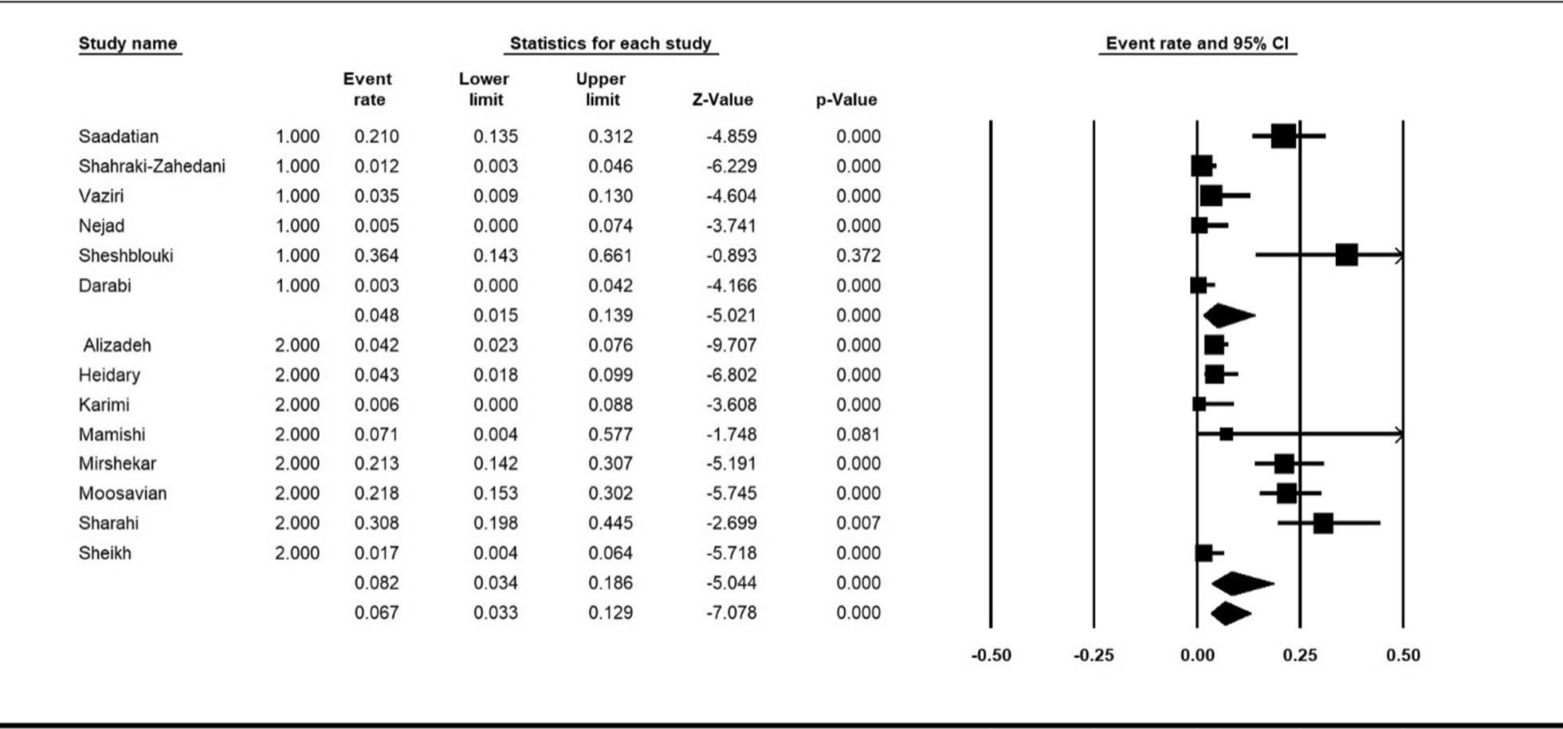
Subgroup meta-analysis for publish year (1: 2013–2018, and 2: 2019–2021)

**Fig. 6:**
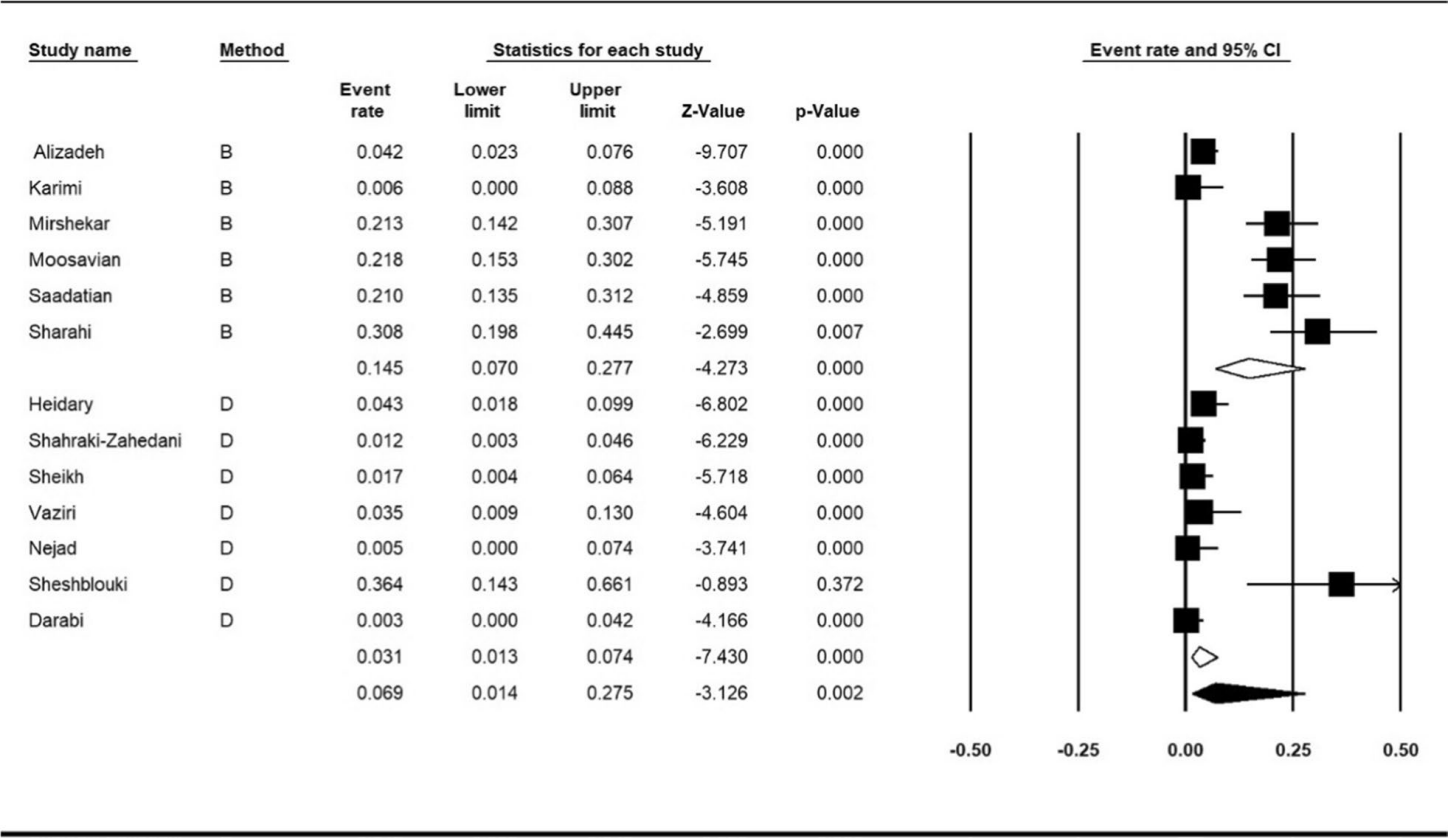
Subgroup meta-analysis for detection method (B: broth microdilution method and D: disk diffusion method)

Subgroup meta-analysis for publish year was performed in two periods (2013–2018 and, 2019–2021). The year subgroup analysis indicated increase resistance of colistin from 4.8%; (95% CI 1.5–13.9%) in 2013–2018 to 8.2%; (95% CI 3.4–18.6%), (I^2^ = 87.12%; *P* < 0.001) in 2019–2021 (Fig. 5). Subgroup meta-analysis based on method of detection antimicrobial resistance revealed 14.5%; (95% CI 7–27%) for broth microdilution method and 3.1%; (95% CI 1.3–7.4%) for disk diffusion method (I^2^ = 88.08%; *P* < 0.001) (Fig. 6).

**Fig. 7:**
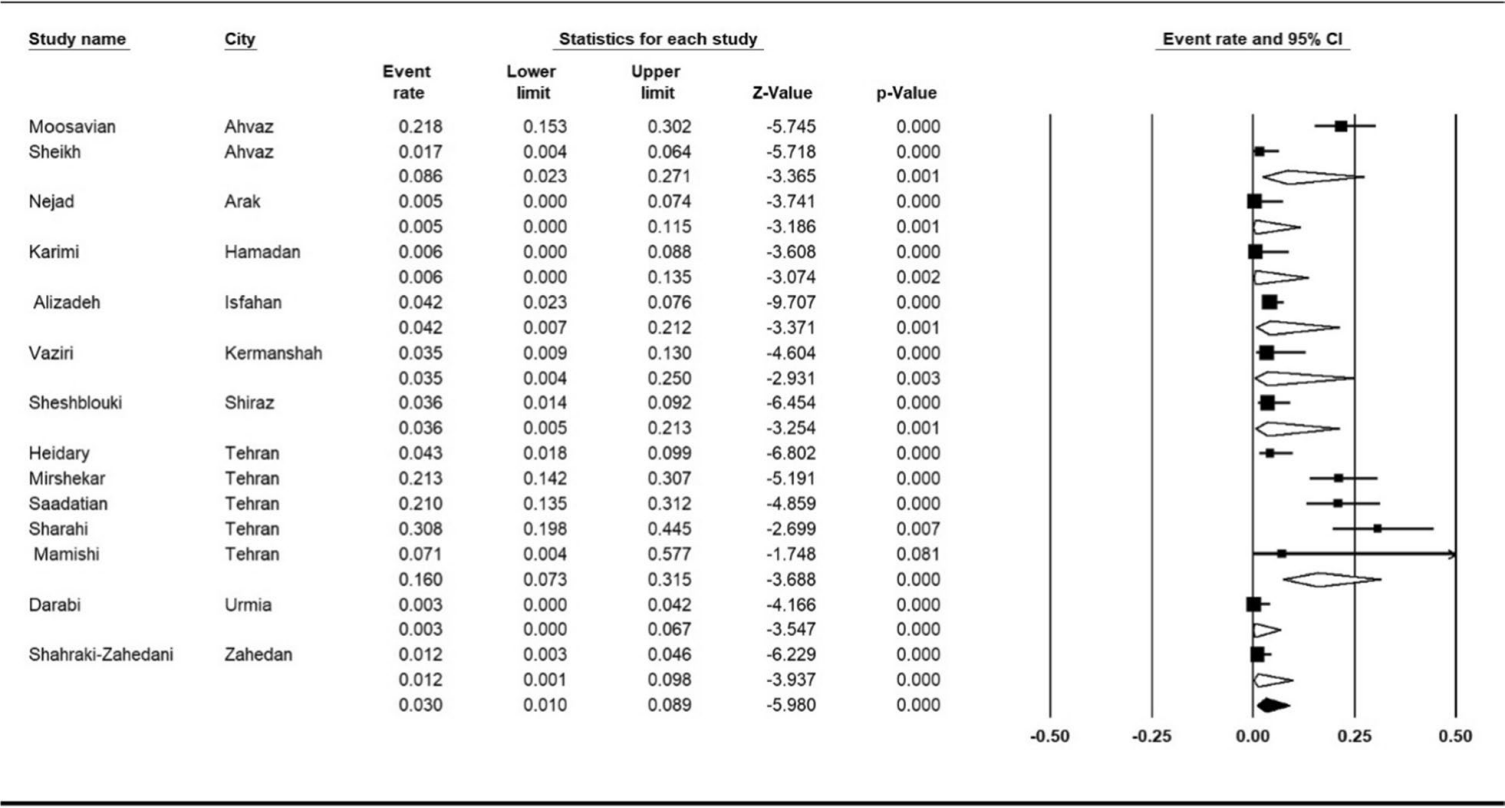
Subgroup meta-analysis for cities

Subgroup meta-analysis of cites showed that Tehran with 16% (95% CI 7.3–31.5%) had the highest resistance to colistin and Urmia with 0.3% (95% CI 0.0–4.2%) had the lowest (I^2^ = 87.61%; *P* < 0.001) (Fig. 7).

**Fig. 8:**
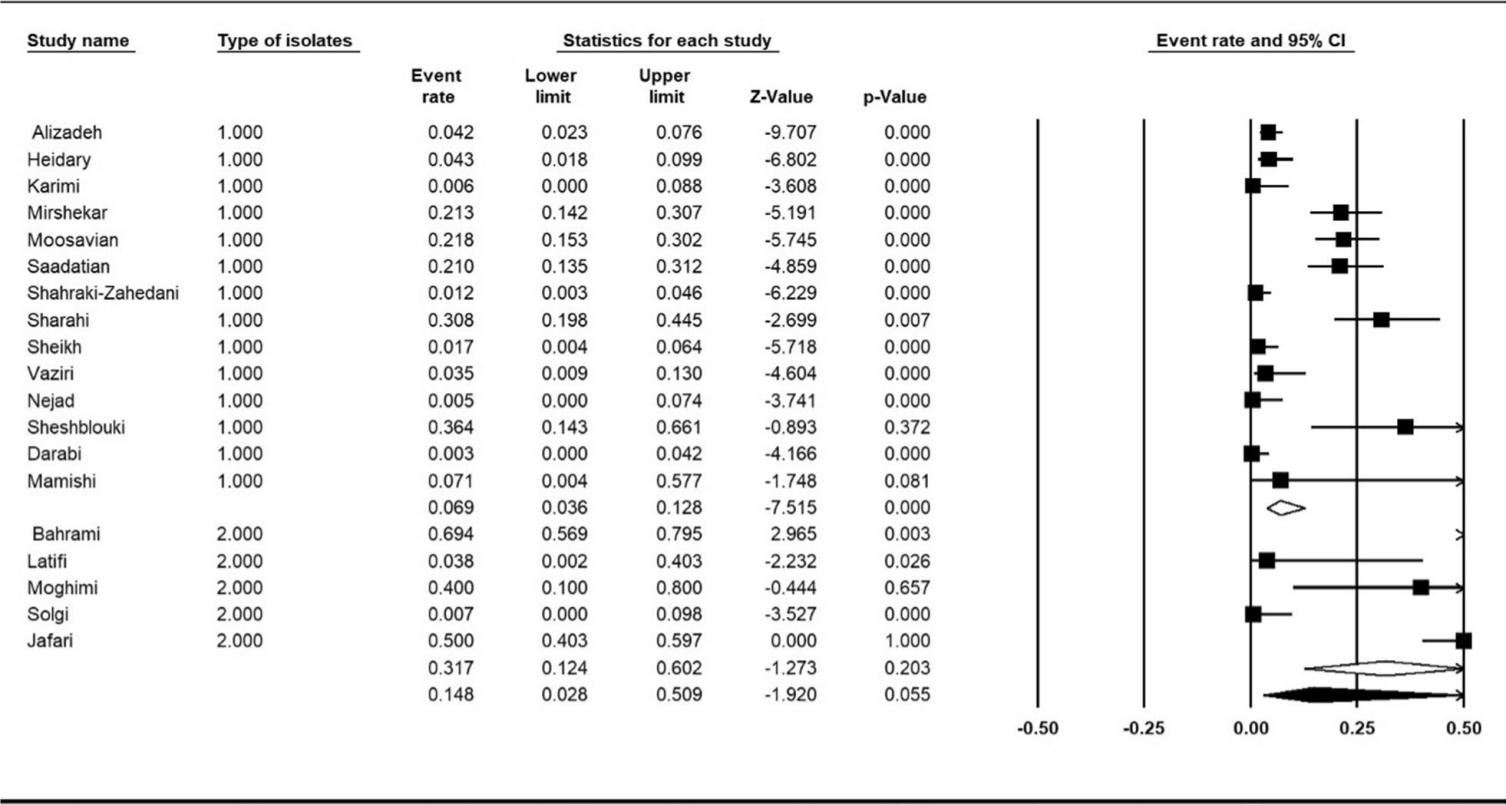
Subgroup meta-analysis based on isolates (1: sensitive *K. pneumoniae* isolates, 2: carbapenemase-producing *K. pneumoniae* isolates)

Five of the included studies reported the prevalence of colistin resistance in Carbapenemase-producing *K. pneumoniae* isolates. We compared the prevalence of colistin resistance in this group of isolates with sensitive isolates. The comparison of resistance in carbapenemase-producing *K. pneumoniae* isolates with sensitive isolates showed a higher level of resistance in carbapenemase-producing *K. pneumoniae* isolates 31.7% (95% CI 12.4–60.2%) compared to sensitive isolates 6.9% (95% CI 3.6–12.8%), (I^2^ = 92.15%; *P* < 0.001) (Fig. 8).

## Discussion

*Klebsiella pneumoniae* is an important opportunistic bacterium that causes severe nosocomial infections [8]. Currently, excessive and inappropriate use of antibiotics against microbial infections has led to increased drug resistance [4, 17]. This resistance raises concerns about the choice of effective antibiotics to treat associated infections. In the past, the use of colistin was limited due to its toxicity, and in the 1970s it was replaced by antibiotics that were considered less toxic. Recently, colistin has been increasingly used as a rescue therapy alone or in combination with one or more other antimicrobials to treat carbapenem-resistant and MDR Gram-negative bacteria [18]. The use of colistin as an option to treat infections caused by carbapenem-resistant and MDR Gram-negative bacteria has led to increased resistance to this antibiotic in recent years [19].

In our research, the pooled prevalence of colistin resistance in clinical *K. pneumoniae* isolates was 6.9% in Iran. A study by Abdelhamid et al. reported that all 50 studied isolates from Egypt were sensitive to colistin [20]. A study by Bshabshe et al. from Saudi Arabia showed 34.5% resistance to colistin [21]. Poudyal et al. In Australia examined 21 MDR *K. pneumoniae*, of which 6 isolates were resistant to colistin (28.5%) [22].Other studies from India, Poland, Pakistan, and Spain reported 16, 0, 4.8 and 13% colistin resistance in *K. pneumoniae* isolates [23–26]. Although a similar meta-analysis is needed to compare the resistance of colistin to other countries, the study of resistance to this antibiotic in Iran showed a moderate resistance level compared to other countries. The results of our study also showed that the rate of colistin resistance increased significantly over time (2013–2018, 2019–2021), which may be due to the increased use of this antibiotic in recent years. Moreover, this increase emphasizes the need to design an accurate program to measure the resistance level to colistin. To measure the resistance to colistin CLSI guideline validates the broth microdilution, disk elution and agar dilution methods, and EUCAST guideline only suggests the microdilution method [27, 28]. Several included articles in this meta-analysis used the disk diffusion method to measure resistance, which is not recommended by EUCAST and CLSI guidelines. The results of the subgroup meta-analysis based on the measurement method showed that the rate of resistance in the disk diffusion method (3.1%) was lower than the broth microdilution method (14.5%), which may indicate that the disk diffusion method may not be sensitive enough to measure and identify resistant strains. Thus, it is essential to use the methods recommended by the standard guidelines.

Although colistin currently maintains a high activity level against most *K. pneumoniae* isolates, the decrease in activity against carbapenem-resistant isolates is worrisome. It has also been recognized that increased colistin use is responsible for outbreaks caused by species intrinsically resistant to polymyxins and the increasing isolation of colistin-resistant *K. pneumoniae* strains. Investigators have reported a correlation between the use of colistin to treat infections caused by carbapenem-resistant and the subsequent emergence of colistin-resistant strains [29]. Mansour et al. In Tunisia isolated 29 carbapenem-resistant *K. pneumoniae*, of which 7 (24.1%) were colistin-resistant isolates [30]. Our study showed a strong association between the carbapenem producing *K. pneumoniae* and increased resistance to colistin. Based on the higher percentage of colistin resistance observed among *K. pneumoniae* isolates producing a carbapenemase (31.7%), it will be necessary to monitor the use of colistin continually.

The present study had a few limitations. The evaluation of antibiotic resistance in *K. pneumoniae* was based on two methods, and this variety of methods can increase heterogeneity. Also, the heterogeneity between studies is relatively high. We used subgroup analysis to discover sources of heterogeneity and reduce the effect of heterogeneity on the results.

## **Conclusion**

Given the recent use of colistin as a life-saving treatment for carbapenem-resistant and MDR *K. pneumoniae*, it is essential to know the prevalence of resistance to this antibiotic. Our study was the first study to investigate the resistance of colistin in *K. pneumoniae* isolates in Iran. The results of our meta-analysis showed a 6.9% prevalence of colistin among the clinical isolates of *K. pneumoniae* in Iran. Our findings support the idea that regulatory measures are essential for the use of colistin.

## Supplementary Information

**Additional file 1.** Search syntax.

## Data Availability

All the data in this review are included in the manuscript.
