# Supplementary material for: Prevalence of colistin resistance of Klebsiella pneumoniae isolates in Iran: a systematic review and meta-analysis
Source: Ann Clin Microbiol Antimicrob. 2022 Jun 28;21:29. doi: 10.1186/s12941-022-00520-8 (PMC9241315; doi:10.1186/s12941-022-00520-8)
Supplement: Supplementary file 1 — Additional file 1. Search syntax. [file 12941_2022_520_MOESM1_ESM.docx]

**Search syntax:**

**PubMed:**

(“Klebsiella pneumoniae” OR K.pneumoniae ) AND (Resistan* OR suscep*) AND (Colisticin OR “Polymyxin E” OR Colimycin OR colistin OR colistimethate) AND Iran.

**Scopus:**

(“Klebsiella pneumoniae” OR K.pneumoniae ) AND (Resistan* OR suscep*) AND (Colisticin OR “Polymyxin E” OR Colimycin OR colistin OR colistimethate) AND Iran.

**Google scholar:**

Klebsiella pneumoniae+ Colistin + Iran.

**SID:**

کلبسیلا پنومونیه
